# Mutations in spliceosome genes and therapeutic opportunities in myeloid malignancies

**DOI:** 10.1002/gcc.22784

**Published:** 2019-09-03

**Authors:** Justin Taylor, Stanley C. Lee

**Affiliations:** ^1^ Human Oncology and Pathogenesis Program Memorial Sloan Kettering Cancer Center New York New York; ^2^ Leukemia Service, Department of Medicine Memorial Sloan Kettering Cancer Center New York New York

**Keywords:** myeloid leukemia, RNA splicing, SF3B1, SRSF2, U2AF1

## Abstract

Since the discovery of RNA splicing more than 40 years ago, our comprehension of the molecular events orchestrating constitutive and alternative splicing has greatly improved. Dysregulation of pre‐mRNA splicing has been observed in many human diseases including neurodegenerative diseases and cancer. The recent identification of frequent somatic mutations in core components of the spliceosome in myeloid malignancies and functional analysis using model systems has advanced our knowledge of how splicing alterations contribute to disease pathogenesis. In this review, we summarize our current understanding on the mechanisms of how mutant splicing factors impact splicing and the resulting functional and pathophysiological consequences. We also review recent advances to develop novel therapeutic approaches targeting splicing catalysis and splicing regulatory proteins, and discuss emerging technologies using oligonucleotide‐based therapies to modulate pathogenically spliced isoforms.

## 1 INTRODUCTION

In 1977, Sharp, Roberts and colleagues discovered that eukaryotic genes are not contiguous but rather “split” by intervening sequences known as introns that are later removed to produce mature messenger RNAs by a macromolecular structure called the spliceosome.^1^, ^2^ One reason introns may have evolved is to diversify the number of messenger RNA species, and subsequently proteins, that can be produced by a single gene through alternative splicing.^3^ As with many other essential cellular processes, cancer cells have co‐opted alternative splicing to promote their survival and response to therapy. Many studies have revealed global dysregulation of splicing in cancer.^4^, ^5^, ^6^, ^7^ For example, synonymous mutations occurring in consensus splice sites can alter intron recognition leading to intron retention and tumor suppressor inactivation.^8^, ^9^ Additionally, genes that encode for regulators of pre‐mRNA splicing are often overexpressed in cancer and may presumably enhance processing of transcripts that are important for cancer cell growth and survival.^10^, ^11^, ^12^ A more extensive review of this literature can be found here.^13^, ^14^

Within the last decade, somatic mutations in genes encoding splicing factors themselves have been discovered at high frequency in patients with hematologic malignancies as well as in epithelial tumors, albeit less commonly.^15^, ^16^, ^17^, ^18^, ^19^, ^20^, ^21^, ^22^, ^23^ Approximately 60% of patients with myelodysplastic syndromes (MDS)^15^, ^16^, ^18^ or chronic myelomonocytic leukemia (CMML), and ~55% of secondary acute myeloid leukemia (s‐AML)^24^ have mutations in genes encoding components of the spliceosome.^15^, ^25^, ^26^ The most common mutations occur in *SF3B1*, *SRSF2*, *U2AF1*, and *ZRSR2* and they tend to be mutually exclusive with one another.^15^ In chronic lymphocytic leukemia (CLL), mutations in *SF3B1* occur in ~15% of patients. Spliceosomal mutations have also been discovered in a number of solid tumors including breast cancer,^27^ pancreatic cancer,^28^ lung cancer,^21^, ^29^ and uveal melanoma.^19^, ^22^, ^30^ With the exception of *ZRSR2* mutations, somatic mutations in *SF3B1*, *SRSF2*, and *U2AF1* cause characteristic changes in pre‐mRNA splicing that are distinct from loss‐of‐function, and will be discussed in greater detail in this article. These mutations are presumed to contribute to oncogenic transformation, but the underlying mechanisms remain elusive and are currently an area of intense research. The high frequency of spliceosomal mutations in myeloid malignancies has generated enthusiasm for their therapeutic targeting and preclinical studies have provided evidence that they can serve as an Achilles' heel in these cancers when using small molecule spliceosomal inhibitors.^31^, ^32^ There is anticipation that a deeper understanding of the oncogenic mechanisms of these mutations might shed light on other therapeutic avenues as well. Last, given the widespread aberrant splicing observed in cancers without spliceosome factor mutations, there is optimism that modulating the activity of the spliceosome may have broader therapeutic applicability in a larger group of cancer patients.

## 2 SPLICEOSOME MUTATIONS IN CLONAL HEMATOPOIESIS AND MYELOID MALIGNANCIES

In 2011, several groups reported high frequency of splicing factor mutations in MDS, most frequently in *SF3B1*, *SRSF2*, *U2AF1*, *and ZRSR2*.^15^, ^16^, ^18^ This striking finding was accompanied by the observation that these mutations were generally mutually exclusive from one another, suggesting they may share overlapping function, and/or are synthetically lethal when coexpressed. Taken together, spliceosomal mutations appears to occur predominantly in MDS patients. Subsequently, whole exome sequencing of 200 de novo AML samples in the Cancer Genome Atlas (TCGA) project^33^ failed to detect any mutations in splicing factors. However, more recent studies have detected spliceosomal mutations occurring in AML using targeted sequencing panels with deeper coverage.^34^ It is possible that splicing factor mutations were not detected among the TCGA cohort due to their low frequency in de novo AML and with the small number of patients they were simply not included or potentially these were missed by mutation calling algorithms due to the high GC content seen in splicing factor genes and the lower depth of sequencing by whole exome sequencing.

Indeed, a more recent study including 1540 patients with AML performed targeted sequencing of 111 genes and cytogenetic analysis and classified 11 subgroups,^34^ including ~18% of AML patients with mutations in chromatin modifiers (*ASXL1*, *STAG2*, *BCOR*, *MLL* ^PTD^, *EZH2*, and *PHF6)* and spliceosome genes (*SF3B1*, *SRSF2*, *U2AF1*, and *ZRSR2*). The chromatin‐spliceosome group was the second largest subgroup (following *NPM1*‐mutant AML, ~27%), and is generally composed of older patients with lower white blood cell counts, lower percentage of blasts, decreased responsiveness to chemotherapy, and an overall poorer survival. The Bayesian statistical model used to derive these subgroups was also applied to the TCGA dataset and found relatively equivalent frequency of subgroups, suggesting a biologically relevant distinction of these classes. The older patients with chromatin‐spliceosome gene mutations appear to be genetically and biologically different from many other subclasses of AML and do not benefit from current treatment paradigms.

Genetically, the chromatin‐spliceosome subgroup of AML resembles a mutation pattern more commonly seen in MDS. It is possible that AML patients that have chromatin‐spliceosome mutations may have had a prodromal MDS period even if they did not necessarily meet the formal criteria for AML with myelodysplasia‐related changes (AML‐MRC).^35^ In fact, a study of 194 patients with rigorously defined s‐AML found that mutations in *SF3B1*, *SRSF2*, *U2AF1*, *ZRSR2*, *ASXL1*, *EZH2*, *BCOR*, or *STAG2* was >95% specific for the diagnosis of s‐AML.^24^ When mutations in these genes were found in de novo AML, they conferred the same poor prognosis as seen in s‐AML. Furthermore, mutations in these genes have also been detected in elderly individuals with clonal hematopoiesis.^36^, ^37^, ^38^ Thus, either as a response to a stressor (genetic or environmental) that accumulates with age or as a phenomenon of aging itself, acquisition of mutations in chromatin modifiers and splicing factors predispose individuals to further development of MDS and/or AML.

A pair of recent studies using large cohorts of individuals with clonal hematopoiesis to attempt to define the factors that associate with progression to leukemia identified that in addition to *TP53* mutations, spliceosomal mutations were associated with high risk of progression to AML.^39^, ^40^ These studies suggest that spliceosomal mutations are among the first mutations to occur in hematopoietic stem cells and because of their very high risk association with leukemia may be disease‐initiating mutations. In fact, genetically engineered mouse models expressing splicing factor point mutations from the endogenous mouse locus provide more evidence to support this hypothesis. Therefore, targeting these mutations might provide the best means to eradicate disease‐initiating cells.

In this review, we will focus on mutations affecting core components of the spliceosome and how they may be targeted for therapeutic applications. This includes mutations in *SF3B1*, *SRSF2*, *U2AF1*, and *ZRSR2*. The first three (*SF3B1*, *SRSF2*, and *U2AF1*) are all components of the major spliceosome and have the unique features of: (a) always occurring as heterozygous change‐of‐function mutations and (b) generally occurring in a mutually exclusive manner. Mutations in *ZRSR2* do not always follow the same pattern; likely because ZRSR2 is not required for major splicing and is primarily a component of the minor spliceosome. These mutations are usually seen as loss‐of‐function and can, on rare occasions, co‐occur with other splicing factor mutations. In the following sections, we will review basic mechanisms of splicing, how mutations in splicing factors affect normal splicing and the potential functional role of these mutations in myeloid neoplasms. We will then discuss the potential for targeting these mutations or reversing their effects with splicing modulators in cancer.

## 3 BACKGROUND ON SPLICING

RNA splicing is a highly coordinated process that removes the intronic portions from the pre‐mRNA and subsequently ligates the protein coding sequences (exons) in the majority of eukaryotic protein coding genes. It is estimated that as many as 95% of human multiexon genes undergo alternative splicing, which can significantly increase the diversity and function of the human proteome.^3^ Splicing represents a critical posttranscriptional mechanism for regulating gene expression, and is orchestrated by a large, dynamic group of ribonucleoprotein complexes known as the major and minor spliceosome. The major spliceosome, which is responsible for removing the majority of human introns, consists of five small nuclear ribonucleoproteins (snRNPs): U1, U2, U4, U5, and U6, while the U5, U11, U12, U4atac, and U6atac snRNPs make up the minor spliceosome.^41^, ^42^ Significant work completed over the last few decades has deepened our understanding of the biochemical composition, regulation and activation of splicing catalysis, and more recently high‐resolution structures of various stages of the spliceosome life cycle have provided detailed insights into this process.^43^, ^44^, ^45^, ^46^ In addition to removing intronic sequences, pre‐mRNA splicing has evolved to couple with other key regulatory pathways involved in gene regulation (reviewed here^47^, ^48^, ^49^).

Splicing catalysis is initiated when defined *cis*‐elements in the pre‐mRNA interact with various *trans*‐acting factors to assemble the spliceosome complex. The catalytic core of the spliceosome is assembled de novo in a series of regulated steps and conformational changes. The U1 snRNP first recognizes the 5′ splice site (GU dinucleotides) via base pairing with its cognate U1 snRNA. Splicing factor 1 (SF1) initially binds to the branchpoint sequence (BPS) located proximal to the 3′ splice site (AG dinucleotides). The U2 auxiliary factors (U2AFs), which form the U2AF1/2 heterodimer complex recognizes the 3′ splice site. The distinction of the 3′ splice site is further reinforced by a stretch of pyrimidine sequences located between the 3′ splice site and the BPS known as the polypyrimidine tract, which serves as an essential signal for recruiting splicing factors to the 3′ splice site. Following the establishment of these factors to form the early complex (Complex E), the U2 snRNP displaces SF1 at the branchpoint via base pairing with U2 snRNAs and interacts with the U2AF complex to form Complex A. The U4/U5/U6 tri‐snRNP is then recruited (pre‐B Complex) to form the activated spliceosome (B* Complex) via conformational rearrangement. The intronic region is then removed and the exons are joined via two sequential trans‐esterification reactions (Complex C). At the completion of splicing catalysis, the spliceosome components and the intron lariat dissociate away from the ligated exons.

The outcome of splicing reactions can be further modulated by *trans*‐acting factors subject to different cellular contexts. For examples, members of the serine/arginine (SR) family of proteins generally possess the ability to promote splicing by sequence‐specific recognition of *cis*‐elements in the pre‐mRNA known as exonic or intronic splicing enhancers (ESE and ISE). Another group of proteins, the heterogeneous nuclear ribonucleoproteins (hnRNPs), are known repressors of splicing by interacting with exonic and intronic splicing silencers (ESS and ISS). These splicing modulators are critical in ultimately dictating the choice of splice site usage, and are extensively reviewed here.^50^

## 4 EFFECTS OF SOMATIC MUTATIONS IN SPLICING FACTORS

**Figure 1:**
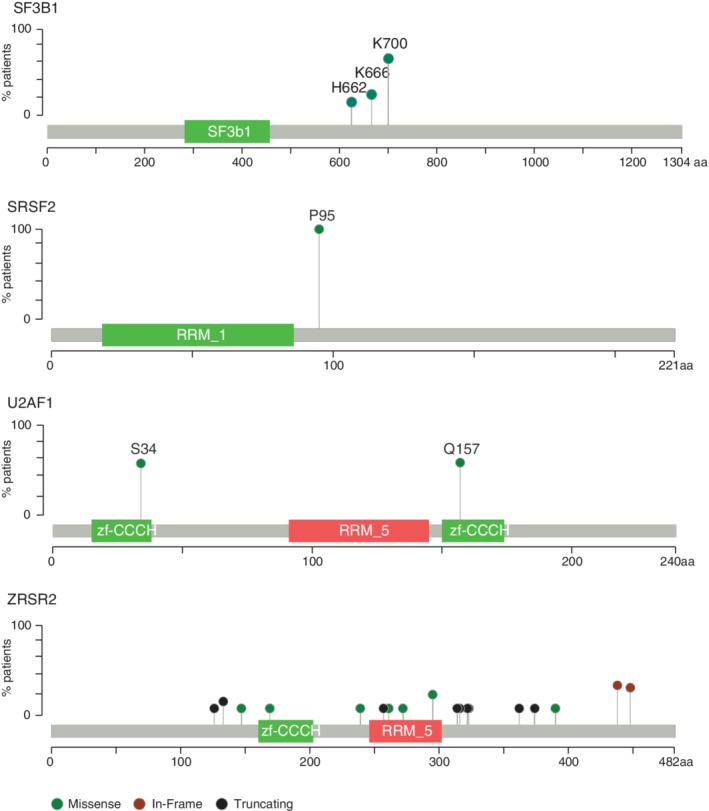
Somatic mutations in major spliceosome associated proteins *SF3B1*, *SRSF2*, and *U2AF1* occur at hotspot residues and mutations in the minor spliceosomal‐associated *ZRSR2* occur as loss‐of‐function across the gene. Lollipop plots showing the frequency of mutations occurring in *SF3B1*, *SRSF2*, *U2AF1*, and *ZRSR2*. The height of the lollipop represents the frequency of occurrence of mutations at the specific residue in that gene as a percentage of all mutations occurring in that gene across myeloid malignancy patients. Green dots represent missense mutations. Red dots represent in‐frame insertions/deletions and black dots represent truncating frameshift or nonsense mutations. Data are from cBioPortal (http://www.cbioportal.org)

One of the most surprising findings from cancer genome sequencing efforts was the identification of recurrent somatic mutations in genes encoding pre‐mRNA splicing factors in both hematologic malignancies including MDS, AML, and CLL,^15^, ^16^, ^17^, ^18^, ^20^, ^51^ and in epithelial cancers such as uveal melanoma, lung adenocarcinoma, breast cancer, and pancreatic ductal adenocarcinoma.^19^, ^21^, ^22^, ^27^, ^30^, ^52^ While mutations have been observed in a large number of spliceosomal genes, *SF3B1*, *SRSF2*, *U2AF1*, and *ZRSR2* are the four most commonly mutated genes (Figure 1). These observations suggest an association between spliceosome gene mutations and a potential role in oncogenesis. Mutations in *SF3B1*, *SRSF2*, and *U2AF1* occur exclusively as heterozygous missense mutations located to very restricted regions, whereas *ZRSR2* mutations are scattered across the gene and are predicted to be loss‐of‐function mutations. Even though the spliceosome machinery contains more than 150 proteins, exactly why only four of these proteins are frequent targets of somatic mutations in hematologic malignancies remains an open question. In the past few years, a wealth of transcriptomic data and functional studies have shed light on the effects of mutant splicing factors on pre‐mRNA splicing and gene expression, and how dysregulation of various target genes can drive disease mechanisms unique to different subtypes of myeloid neoplasms.

### 4.1 SF3B1 mutations

In hematologic malignancies, *SF3B1* mutations are commonly found in MDS,^16^, ^53^ AML, myeloproliferative neoplasms (MPN) and in some MDS/MPN overlap syndromes,^54^ and in ~10%‐15% of CLL patients.^17^, ^20^ Mutations in *SF3B1* are specifically enriched in a subtype of MDS previously known as refractory anemia with ring sideroblasts (RARS), characterized by anemia and dysplastic erythroblasts with abnormal iron accumulation in the mitochondria^55^ causing a “ring” of blue granules to appear around the nucleus upon Prussian blue staining. RARS has been renamed MDS with ring sideroblasts (MDS‐RS) and is generally associated with a favorable clinical course.^16^, ^53^ Interestingly, *SF3B1* mutations are so common in MDS‐RS that the WHO classification criteria have recently been revised to allow diagnosis of MDS‐RS with as low as 5% ring sideroblasts in the presence of mutant *SF3B1*.^35^ Most of the mutations in *SF3B1* are clustered near the HEAT repeat domains 4 to 7 (HR4‐HR7), with the most frequently mutated residues being K700 and K666 in MDS and CLL; while mutations in the R625 position are the most commonly occurring allele in uveal melanoma. The functional relevance of these distinct mutations to disease subtypes still remains unclear, and is an interesting area of focus for future studies.

**Figure 2:**
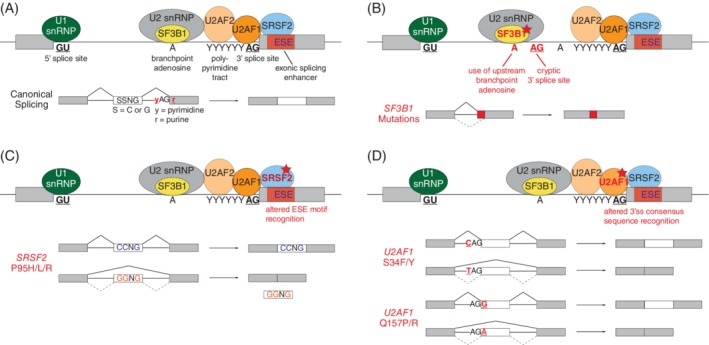
Functional consequences of *SF3B1*, *SRSF2*, and *U2AF1* mutations in on RNA splicing. A, A description of canonical splicing. B, Somatic mutations (marked by a red star) in *SF3B1* result in enhanced usage of cryptic 3′ splice sites ~10‐40 nucleotides upstream of the canonical 3′ splice site (marked by black AG dinucleotide), and recognition of an upstream adenosine residue by the U2 snRNP/SF3B1 complex. This results in the aberrant incorporation of intronic sequences in the final transcript. C, *SRSF2* mutations are clustered at the proline 95 residue, and alter the preference of exonic splicing enhancer (ESE) motif recognition. Usually, wildtype SRSF2 have equal preference for CCNG and GGNG ESE motifs in cassette exon splicing. Mutant SRSF2 show preferential inclusion of exons with CCNG‐containing motifs over GGNG‐containing motifs. D, Mutations in *U2AF1* alter the 3′ splice site usage that is dictated by sequences flanking the intron‐exon junction (the AG dinucleotide). In the presence of *U2AF1* S34F/Y mutation, exons are preferentially included if the pyrimidine preceding the AG dinucleotide (ie, the “–3” position) is a C instead of a T. Similarly, in *U2AF1* Q157P/R mutant cells, this preference is determined by the purine nucleotide immediate after the AG dinucleotide (ie, the “+1” position), such that a G instead of A results in preferential exon inclusion

SF3B1 is a component of the U2 snRNP that binds to the branchpoint during the formation of Complex A, and is predicted to be ubiquitous in recognizing the majority of 3′ splice sites.^56^ Transcriptomic analyses have revealed that the major splicing defect associated with *SF3B1* mutations, regardless of cellular or disease origin, is the preferential usage of cryptic 3′ splice sites approximately 10‐30 nucleotides upstream of the canonical 3′ splice site (Figure 2). This is distinct from loss‐of‐function of SF3B1 that causes inefficient splicing catalysis.^57^, ^58^, ^59^ The region around the cryptic 3′ splice site coincides with an enrichment of adenosines that also appear to have stronger base‐pairing affinity with the cognate U2 snRNA relative to the region around the canonical BPS. Structural analyses of the human and yeast spliceosome in the RNA‐bound B‐activated Complex suggest that cancer‐associated mutations in *SF3B1* change the charge and shape of the corresponding amino acid residues that results in direct disruption of the local interaction with pre‐mRNA.^46^, ^60^, ^61^, ^62^ This results in a spatial shift in the pre‐mRNA by approximately 10 nucleotides, consistent with bioinformatic predictions.

A study from Darman et al predicted that mutant SF3B1‐induced cryptic 3′ splice site usage can introduce premature termination codons that are subjected to nonsense‐mediated decay (NMD) in approximately half of the aberrantly spliced transcripts.^58^ Some of the most well defined targets of this NMD such as ABCB7, SLC25A37, TMEM14C, and ALAS2 have provided some insights into a potential causal link between *SF3B1* mutations and MDS‐RS. *SF3B1* mutation induces mis‐splicing of ABCB7 by aberrant 3′ splice site usage that results in NMD, and subsequent downregulation of ABCB7 mRNA and protein. ABCB7 is a mitochondrial iron exporter responsible for maintaining iron homeostasis,^63^ and there is a strong correlation between MDS‐RS and dysregulation of ABCB7.^64^, ^65^ SLC25A37 is also another iron transporter mis‐spliced by mutant SF3B1. Mice engineered to inducibly express *Sf3b1*K700E mutation at physiologic level^66^, ^67^ exhibited mild defects in erythropoiesis, but did not develop RS. More importantly, Abcb7 mis‐splicing was not observed in either of the mouse models due to lack of species conservation of the intronic sequences. Other MDS‐RS associated genes such as *Tmem14c*, *Alas2*, and *Slc25a37*, were not aberrantly spliced or expressed in *Sf3b1*K700E mice.^66^ Additionally, mice lacking *Abcb7* did not develop any RARS‐associated phenotypes^65^; therefore, it is possible that other aberrant splicing events in addition to that in ABCB7 are required to drive MDS‐RARS. Overall, while global transcriptomic studies are powerful tools for inferring direct targets of aberrant splicing, these studies also highlight current challenges associated with identifying the functionally relevant and causative mis‐splicing events that drive specific disease phenotypes.

### 4.2 SRSF2 mutations

*SRSF2* mutations are found commonly in ~50% of CMML, ~15% of MDS, ~20% of s‐AML patients, and are often associated with poor prognosis and a higher risk of transformation to acute leukemia.^26^, ^51^, ^68^, ^69^ SRSF2 is a member of the serine/arginine‐rich (SR) family of proteins that recognizes specific RNA motifs, and is generally a positive regulator of exon inclusion.^70^ Somatic mutations in *SRSF2* are restricted to the P95 amino acid residue near the RNA recognition motif. Extensive analysis using primary patient materials and complementary model systems revealed that the amino acid substitution at the proline 95 position alters the RNA binding activity of SRSF2 in a sequence specific manner. Normally, canonical SRSF2 recognizes and binds to C‐rich and G‐rich motifs in the ESE with similar affinity.^71^, ^72^ In contrast, mutant SRSF2 protein preferentially binds C‐rich motifs and thus suppresses the inclusion of G‐rich containing exons, resulting in widespread changes in splicing (Figure 2). Several prominent targets include chromatin modifiers EZH2 (inclusion of a “poison” exon that induces NMD and global transcript downregulation), BCOR (cassette exon splicing), CASP8 (cassette exon splicing that results in generation of a novel truncated isoform^73^), and FYN (mutually exclusive exons).^72^, ^74^ Interestingly, *EZH2* loss‐of‐function mutations and *SRSF2* missense mutations are highly mutually exclusive in MDS patients, and the ability of mutant SRSF2 to suppress EZH2 may represent a mechanistic explanation for this observation.^53^, ^68^

### 4.3 U2AF1 mutations

*U2AF1* mutations are found in ~15% of MDS patients without RARS, in ~10% of CMML, ~10% of s‐AML,^15^, ^18^, ^68^ ~10% of hairy cell leukemia variant (HCL‐v),^75^ and is generally associated with poor prognosis, and increased risk of leukemic transformation. It is also found in a subset of pancreatic ductal adenocarcinomas^28^ and nonsmall cell lung adenocarcinomas.^21^ U2AF1 normally recognizes the AG‐dinucleotide at the 3′ splice site during the early steps of splicing catalysis in a sequence‐specific manner.^76^ Mutations in *U2AF1* are concentrated on two distinct amino acid residues, S34 and Q157, both of which are located within the two distinct zinc finger domains. RNA‐seq analyses from human patient samples revealed that *U2AF1* mutations induces aberrant splicing of ~5% of total transcripts predominantly via aberrant cassette exon skipping or inclusion, and to a lesser extent, alternative 3′ splice site usage. The exact splicing pattern mediated by mutant U2AF1 is dictated by consensus sequences around the AG‐dinucleotide at the 3′ splice site. The S34 mutation generally promotes cassette exon inclusion if the nucleotide immediately preceding the AG‐dinucleotide is C/A over T (ie, the “−3” signature), whereas the Q157 mutation preferentially includes exons containing G over A immediately after the AG‐dinucleotide (ie, the “+1” signature; Figure 2).^29^, ^77^, ^78^, ^79^ Direct mis‐spliced targets include H2AFY, BCOR, ATR, FANCA, STRAP, CASP8, PICALM, and GNAS. Functional studies using human CD34^+^ cells from the cord blood expressing aberrant isoforms of H2AFY and STRAP resulted in skewed myelomonocytic differentiation at the expense of erythroid differentiation that phenocopies *U2AF1* S34F cells, suggesting a potential causal link.^80^ Moreover, pathway analysis revealed key signatures are dysregulated by *U2AF1* mutations, including DNA damage response, pre‐mRNA splicing, RNA localization and transport, epigenetic regulation and cell cycle.^29^, ^77^, ^79^, ^81^

### 4.4 ZRSR2 mutations

*ZRSR2* mutations are found in ~5%‐10% of MDS patients, and are located sporadically across the entire coding region, and are predicted to be loss‐of‐function mutations. Mutations of *ZRSR2* are found predominantly in male patients, most likely because *ZRSR2* is an X‐linked gene.^15^ Usually X chromosome genes are functionally haploid in both men and women due to X‐inactivation in females; however, tumor suppressor genes on the X chromosome have been observed to “escape” inactivation to have biallelic expression in females.^82^ As males have only a single X chromosome, inactivating mutations in tumor suppressors decrease the gene dosage but females are able to maintain a functional copy by escaping X‐inactivation, resulting in a male predominance in cancers related to these X‐linked tumor suppressor genes. ZRSR2 is a core component of the minor spliceosome that is responsible for splicing of minor/U12 introns, which represents less than 1% of all human introns.^42^ Loss of ZRSR2 is predicted to lead to increased retention of U12 introns without affecting U2 intron splicing (Figure 2). Initial work done in vitro identified several mis‐spliced genes involved in cell cycle regulation and MAP kinase signaling pathways.^83^

### 4.5 Functional consequences of splicing factor mutations in pathogenesis of myeloid neoplasms

Insights into the functional consequences of splicing factors alterations in vivo have emerged using isogenic models of genetically engineered mouse models and human cell lines. Several different conditional knock‐in alleles of *Sf3b1*K700E, *Srsf2*P95H, and *U2af1*S34F mutations as well as a tetracycline‐inducible allele of the *U2af1*S34F mutation have been generated to study the effect of hematopoietic‐specific expression of each of the respective splicing mutations.^66^, ^67^, ^72^, ^79^, ^84^, ^85^, ^86^ While there were variations in the targeting strategies during the constructions of these mutant alleles,^87^ the overall impact of various splicing factors mutations on the hematopoietic system were generally similar (summarized here^88^, ^89^). In the setting of native hematopoiesis, expression of *Srsf2*P95H and *U2af1*S34F mutations resulted in mild leukopenia, and macrocytic anemia in the blood, as well as defects in erythroid and B‐cell differentiation and myeloid skewing in the bone marrow.^72^, ^79^, ^84^, ^85^, ^86^ Mice expressing the *Sf3b1*K700E mutation exhibited erythroid differentiation defect and mild anemia, but these mice did not develop signs of ring sideroblasts.^66^, ^67^ Functional assessment of hematopoietic stem and progenitor cells (HSPCs) by competitive bone marrow transplantation assays showed that mutations in *Sf3b1*, *Srsf2*, and *U2af1* uniformly resulted in reduced repopulating potential compared with wildtype HSPCs.^66^, ^67^, ^72^, ^79^, ^84^, ^85^, ^86^ Similarly, reduced proliferation was also observed in isogenic induced pluripotent stem cells derived from primary MDS patients with spliceosome gene mutations,^71^ and isogenic cancer cells lines.^90^ Less is known about the effects of defective U12 intron splicing driven by *Zrsr2* mutations on hematopoiesis in vivo due to the lack of *Zrsf2* knockout mice. In vitro, the effect of *ZRSR2* depletion by RNAi altered erythroid and myeloid differentiation potential in human CD34^+^ cord blood cells, and resulted in reduced proliferation of leukemia cell lines.^83^ Moreover, a series of genetic and pharmacologic studies have revealed that both hematologic and epithelial malignancies that carry spliceosomal mutations are highly dependent on the wildtype allele for survival,^31^, ^32^, ^67^, ^90^, ^91^, ^92^ and that expression of multiple spliceosome gene mutations using rigorous isogenic models can induce synthetic lethality.^73^ These observations have provided a rationale for designing therapeutic strategies targeting the spliceosome or splicing regulatory proteins in cancer (discussed in detail later). Although these isogenic models recapitulate certain aspects of human MDS based on longitudinal follow‐up studies, none of the splicing factor mutant models developed acute leukemia, suggesting a role for additional cooperating mutations and/or nongenetic factors in disease progression. Nonetheless, these models represent valuable reagents to study altered splicing in leukemogenesis in an isogenic context.

Despite the recent advances gained from modeling mutant splicing factors in vivo, there are several outstanding questions that remain. The reason why distinct splicing factor mutations are enriched in specific cancer subtypes remains unknown. In addition, the mechanistic basis of how splicing factor mutations confer selective advantage during the evolution of MDS pathogenesis remains a mystery. Although recent large‐scale transcriptomic analysis of mutations affecting *SF3B1*, *SRSF2*, and *U2AF1* revealed global splicing dysregulation consistent with their predicted effects, there was minimal overlap between mis‐spliced transcripts altered by each of the mutant proteins.^93^, ^94^ To identify key splicing abnormalities induced by mutant splicing factors, several studies provided functional evidence that expression of the abnormal splice isoforms in normal cells, or reintroduction of the canonical isoforms in spliceosomal mutant cells, can only partially phenocopy or rescue the effects of mutant spliceosome proteins.^72^, ^80^, ^95^, ^96^ These findings suggest that the pathological effects of spliceosome mutations are imparted through multiple mis‐spliced products simultaneously. In parallel, pathway analyses followed by functional validation revealed that splicing factor mutations distinctly converge on key cellular pathways to drive disease pathogenesis, including defects in DNA damage response and aberrant innate immune signaling.^73^, ^93^, ^97^, ^98^ Further studies will be required to systematically address these fundamental questions, and more importantly, to identify the key players and pathways that can potentially be exploited for therapeutic purposes.

## 5 THERAPEUTIC IMPLICATIONS

Prior to the discovery of spliceosome gene mutations in cancer, naturally derived compounds that modulate pre‐mRNA splicing were being tested as anticancer agents in cancer cell lines.^99^, ^100^ These studies showed that cancer cells in general are sensitive to splicing inhibition. At the time, it was unclear why disrupting an essential process like splicing would render cancer cells more vulnerable than their nontransformed counterparts. Three recent studies, mainly in MYC‐driven cancers, have uncovered potential mechanistic explanations. In a forward genetic screen to identify genes required to tolerate aberrant MYC activation, Hsu et al identified that the core spliceosome is a novel dependency in *MYC*‐overexpressing breast cancers that can be targeted therapeutically. This is likely because of increased transcription and production of pre‐mRNA that these cells rely heavily on optimal spliceosome function.^101^ Similarly, Hubert et al identified that *MYC*‐overexpressing glioblastoma stem cells are selectively more susceptible to loss of PHF5A, a component of the U2 snRNP, relative to untransformed neural stem cells.^102^ In the third study using a *MYC*‐driven murine lymphoma model, Koh et al demonstrated that *MYC* overexpression upregulates transcription of genes that encode core components required for assembling the spliceosome complex, including Prmt5, a protein arginine methyltransferase that methylates Sm proteins during U2 snRNP maturation.^103^ These examples highlight a unique feature that cancer cells, at least those overexpressing *MYC*, require a balanced splicing regulatory network for survival. It is tempting to speculate that this principle could be applied to other non‐*MYC*‐driven cancers with increased proliferation and transcription. In this regard, however, the principle of inhibiting splicing might not be considered very different from conventional chemotherapy, where the idea is that highly proliferative cancer cells depend on DNA replication and transcription. However, some other approaches have viewed splicing inhibition as a synthetic lethal approach wherein certain abnormalities, namely splicing factor mutations, might predispose cells to be more sensitive than other cells.^31^, ^32^ Thus, a therapeutic window may exist wherein susceptible cancer cells are sensitive to splicing modulatory drugs at a level that is tolerated by healthy cells.

### 5.1 Targeting the core spliceosome with SF3B modulatory compounds

A little over two decades ago, inhibitors of the U2 snRNP were derived from bacterial species (FR901464/Spliceostatin A from *Pseudomonas* spp.^104^, ^105^ and herboxidienes^106^, ^107^ and pladienolides^108^, ^109^ from *Streptomyces* spp.) originally for use as pesticides and antibiotics. These compounds were identified to have antitumor properties by causing cell cycle arrest in the G1 and G2/M phases and it was not until later that the SF3B component of the U2 snRNP was identified as the primary target.^110^, ^111^, ^112^ Biochemical assays identified that pladienolides and spliceostatin A inhibit splicing by abolishing the interaction between the U2snRNP/SF3B complex to the branchpoint region of the pre‐mRNA.^113^, ^114^ The discovery that a mutation in *SF3B1* R1074H conferred resistance to pladienolides demonstrated that SF3B1 was a direct target of this class of splicing inhibitory compounds.^115^ This was validated in a more recent study that identified a series of acquired mutations in *SF3B1* (K1071 and V1078) in addition to R1074H, and in *PHF5A* Y36C that also conferred resistance to pladienolides.^116^ The crystal structure of pladienolide B bound to the human SF3B complex was recently solved and confirmed that, in addition to SF3B1, pladienolide B also interacts with PHF5A, a PHD‐finger‐like domain containing member of the SF3B complex.^117^ Structural analysis showed that pladienolide B fits into a hinge area that prevents the transition to a closed confirmation and precludes recognition of the branchpoint adenosine. This work is consistent with previous RNA sequencing data that showed intron retention as the major splicing alteration upon exposure to small molecule spliceosome inhibitors.^31^, ^32^ Last, structural analysis also suggested that because of the unique binding pocket and related shape of all the currently described SF3B inhibitors (pladienolides, herboxidienes, and spliceostatin A), this class of inhibitors acts by similar mechanisms.

The first clinical trials with small molecule SF3B inhibitors were performed in unselected epithelial malignancies with E7107, a semisynthetic derivative of pladienolide B. In two separate studies, one in Europe^118^ and one in the United States,^119^ E7107 was tested in a phase 1 dose escalation trial. In the U.S. study, 26 patients were treated and two developed dose‐limiting toxicities at 5.7 mg/m^2^ giving intravenously on days 1 and 8 of 21‐day cycles, including one patient who had a myocardial infarction. The maximum tolerated dose was determined to be 4.3 mg/m^2^ and a total of six patients received that dose. However, one patient developed acute visual loss with a central scotoma and the trial was halted. At the lower dose of 3.2 mg/m^2^, another patient out of four treated at that dose also developed the same pattern of visual loss but to a lesser degree. At that time, the European study was also stopped after treating 40 patients to a maximum dose of 4.0 mg/m^2^ given on days 1, 8, and 15 of a 28‐day cycle. Indeed, one patient on the 4.0 mg/m^2^ dose had started to develop visual symptoms when taken off the drug but still progressed to visual loss with central scotoma; however, the patient ultimately recovered to mild symptoms only. These episodes of vision loss were considered to be related to optic neuritis and potentially related to E7107 toxicity. Even at doses that pharmacodynamically affected splicing, there was only a single partial remission in both of the trials, and an additional 16 patients (35%) had stable disease. Therefore, subsequent efforts focused on developing potentially safer drugs or finding subsets of patients that might respond to lower doses.

In the presence of splicing factor mutations, the idea that cells become dependent on basal splicing and are thus more sensitive to pharmacologic splicing perturbation is an appealing therapeutic proposition. This hypothesis has been tested in vitro and in vivo in preclinical models and in clinical trials.^31^, ^67^, ^90^, ^91^, ^92^ As discussed above, simultaneous preclinical efforts to discover the biology underlying splicing factor mutations in hematologic neoplasms identified that *Srsf2* ^P95H/+^ mutant hematopoietic cells required the wildtype allele of *Srsf2* to survive. In a murine leukemia model driven by MLL‐AF9 fusion oncogene, *Srsf2* ^P95H/+^ leukemias were more sensitive to E7107 than *Srsf2* ^*+/+*^ wildtype leukemias.^31^ Similarly, increased sensitivity to spliceosome inhibitors were also observed in *Sf3b1* ^K700E/+^ and *U2af1* ^S34F/+^ murine hematopoietic cells in vivo,^67^, ^92^ and in *SRSF2*‐mutant CMML patient‐derived xenograft model.^32^ These studies provided proof‐of‐concept that a therapeutic window might exist for SF3B inhibitors to achieve the requisite splicing inhibition to selectively kill spliceosomal mutant cells. This coincided with the development of H3B‐8800, an orally bioavailable derivative of pladienolide B that showed structural similarity to E7107 but with less potency. Further preclinical testing demonstrated that H3B‐8800 also selectively affected splicing factor mutant myeloid neoplasms by causing enhanced retention of GC‐rich introns in splicing factor mutant cells that are enriched in genes encoding spliceosomal proteins themselves.^32^ Ongoing efforts will be described later but a deeper understanding of the molecular targets of known inhibitors and their effects on splicing will help set the stage for this discussion. A phase 1 clinical trial of H3B‐8800 has recently been initiated targeting patients with relapsed/refractory myeloid neoplasms (MDS, CMML, and AML) that carry splicing factor mutations (NCT02841540).

### 5.2 Targeting RNA splicing regulatory proteins

In addition to targeting the core spliceosome using small molecule inhibitors, splicing modulation can also be achieved by targeting proteins involved in regulating the wider splicing network. Recent studies have identified such pathways that have potential clinical and therapeutic applications. First, alterations of splicing regulatory proteins have been observed in diverse cancer subtypes and have become apparent antineoplastic targets. Inhibiting the SRSF2 kinases SPRK and CLK, as well as the SF3B1 kinase DYRK have been linked to effects on splicing factor phosphorylation, localization, and alternative splicing.^70^, ^120^, ^121^, ^122^, ^123^ A large‐scale screen for more selective splicing factor kinase inhibitors discovered three related compounds, Cpd‐1, Cpd‐2, and Cpd‐3 that seem to specifically inhibit SPRK1, SRPK2, and/or CLK1,2 and modulated pre‐mRNA splicing resulting in cell growth suppression and apoptosis.^124^ It remains to be determined if spliceosomal mutant leukemia cells may exhibit differential sensitivity to these splicing modulatory compounds relative to splicing wildtype counterparts. Further preclinical studies of these compounds and of inhibitors of splicing regulatory proteins in general are needed to identify their mechanisms of action on splicing regulation, and verify their full therapeutic potential.

The aforementioned MYC‐PRMT5‐RNA splicing axis suggests that inhibiting PRMT5 and its associated RNA splicing regulatory network may be an attractive and alternative strategy to target spliceosome‐mutant leukemias. The PRMT family of proteins catalyzes arginine dimethylation on histone and RNA binding proteins (RBPs) via the transfer of methyl groups from *S*‐adenosylmethionine to a guanidine nitrogen atom of arginine residues, thereby releasing *S*‐adenosyl‐homocystein as a byproduct.^125^ They can be broadly categorized into type‐I (PRMT1, 2, 3, 4, 6, and 8) and type‐II PRMTs (PRMT5, 7, and 9) that catalyze asymmetric and symmetric arginine dimethylation, respectively. One well‐documented function of PRMTs is the methylation of three Sm proteins (B/B′, D1, and D3) required for the assembly of spliceosome‐related snRNPs. The general function of PRMTs in RNA splicing regulation can be inferred from observation that genetic loss of *Prmt5* caused global splicing alteration in murine neural stem/progenitor cells^126^ and murine hematopoietic stem cells.^127^, ^128^ PRMT5 is overexpressed in a variety of human tumor types and several orally bioavailable PRMT5 inhibitors and, most recently, a novel type‐I PRMT inhibitor are now in early‐phase clinical trials for patients with non‐Hodgkin's lymphoma, advanced solid tumors and in low‐risk MDS (Table 1). Moreover, a recent study suggests that pharmacologic inhibition of type‐I PRMTs also perturbs RNA splicing globally and synergizes with PRMT5 inhibition to induce antitumor effects.^129^ Based on these observations, it would be tempting to speculate that leukemias bearing spliceosome gene mutations may also confer differential sensitivity to PRMT5 and/or type‐I PRMT inhibitors.

**Table 1:** Therapeutic agents and targets currently under development for splicing factor mutant myeloid malignancies

Agent	Target	Preclinical efficacy	Clinical testing
E7107 H3B‐8800	SF3B1	Yes	NCT02841540
Spliceostatin A Meayamycin B Sudemycin C/D	SF3B1	Yes	No
GSK3368715	Type‐I PRMTs	Yes	NCT03666988
GSK3326595 JNJ‐64619178	PRMT5	Yes	NCT02783300 NCT03614728 NCT03573310
Sulfonamides Indisulam E7820	RBM39	Yes	No
Cpd 1 Cpd 2 Cpd 3	SPRK1/2 and CLK1/2	No	No
ASOs morpholinos	Splicing enhancers/silencers	No	No

*Notes*: Similar classes of drugs are grouped together and categorized by their main target. Preclinical efficacy is defined by strong studies in cancer setting relevant to splicing mutant malignancies or characterizing effects of the drug on splicing in cancer. Clinical is defined by clinical trials in cancer patients published on http://www.clinicaltrials.gov.

### 5.3 Targeting RBPs: anticancer sulfonamides and RBM39

Two elegant studies focused on identifying the molecular targets of anticancer sulfonamides have revealed a potential therapeutic application for spliceosomal mutant leukemias. Previous clinical trials have shown that sulfonamides are generally well tolerated, but the observed efficacy in cancer patients was limited.^130^, ^131^, ^132^, ^133^, ^134^, ^135^ Using distinct target identification approaches, the authors concluded that RBM39 is the putative molecular target of sulfonamide compounds including indisulam, E7820, and chloroquinoxaline.^136^, ^137^ Mechanistically, the sulfonamides act as molecular glue by targeting RBM39 to the CUL4‐DCAF15 ubiquitin complex for proteasome degradation in a manner that depends on *DCAF15* expression level. RBM39 is an RNA‐binding protein that has several key roles including transcriptional coactivation and pre‐mRNA splicing. The authors provided evidence that RBM39 depletion resulted in global splicing alterations including preferential cassette exon skipping and intron retention. A recent study by Wang et al confirmed that RBM39 loss resulted in repressed cassette exon splicing and increased intron retention, and that spliceosome‐mutant leukemias showed preferential sensitivity to pharmacologic targeting of RBM39.^138^ These studies have raised the interesting possibility that spliceosome mutant leukemias may be sensitized to anticancer sulfonamide compounds, and is an interesting research area that may potentially motivate future clinical trials.

### 5.4 Targeting splice isoform alterations using oligonucleotide‐based therapy

Another avenue to therapeutically target splicing activity in cancers is the selective targeting of the pathological splicing events with oligonucleotide‐based approaches. The prototype for this class of therapies is the antisense oligonucleotides (ASOs) that have recently been approved in the United States for the treatment of Duchenne muscular dystrophy^139^ and spinal muscle atrophy (SMA).^140^ These oligonucleotides hybridize to RNA and can either target it for degradation or be used to affect splicing of pre‐mRNA. In the case of SMA, the approved oligonucleotide targets an intronic splicing silencer of *SMN2* without degrading the pre‐mRNA and allows for inclusion of an exon that results in increased stability of the protein.^141^ Bifunctional ASOs that are composed of a region complementary to the targeted exon and a noncomplementary region that contains an exonic splicing enhancer sequence can be used to recruit spliceosomal proteins that promote exon inclusion.^142^ These approaches have not yet been successfully applied to cancers but one study using small molecule splicing inhibitors proved that this may be a useful concept in melanomas expressing a *BRAF* V600E splice variant.^143^ Some patients with *BRAF* V600E mutant melanoma treated with the BRAF inhibitor vemurafenib develop resistance by expressing an alternatively spliced form of BRAF that lacks the RAS‐binding domain. A recent preclinical study found that treatment with spliceostatin A restored inclusion of the exons encoding the RAS‐binding domain and reverted the cells back to a vemurafenib‐sensitive state.^144^ This could also conceivably be achieved with ASOs, potentially with less toxicity than global splicing perturbation. Last, in an effort to improve stability of ASOs, synthetic oligonucleotides composed of subunits with a morpholine ring instead of a ribose ring, termed morpholino, was developed.^145^ Morpholino technology seems especially suitable to targeting splicing because they are not recognized by RNAse H and therefore do not cause direct degradation of the pre‐mRNA. In fact, it has been shown that the targeted pre‐mRNAs remain localized to the nucleus and can be visualized using RNA in situ hybridization.^146^ The morpholino oligonucleotides also lack the negatively charged backbone of traditional ASOs that may interact nonspecifically with other components of the cell and thus may be less toxic as a result. Despite these recent technological advances and clinical achievements in neuromuscular disorders, the utility of ASOs in spliceosome mutant leukemias remains challenging, primarily due to our insufficient understanding of the key mis‐spliced targets responsible for disease initiation and/or progression. Future work focusing on systematic identification of pathogenic isoform alterations will be key to exploit the full potential of this therapeutic approach.

## 6 CONCLUSIONS AND FUTURE DIRECTIONS

The field of targeting splicing as an anticancer therapeutic is in its nascence. The major idea leading the field so far and currently being tested in an early phase clinical trial is the use of spliceosomal modulation to induce synthetic lethality in splicing factor mutant malignancies. Whether drugs that target the SF3B complex can achieve this with minimal on‐target toxicity remains to be seen. Regardless, more therapies that globally perturb splicing via alternative mechanisms could be developed into therapeutic approaches. As discussed above, inhibiting regulatory proteins such as splicing regulatory kinases (eg, CLKs and SRPKs) and PRMTs are currently being explored as potential therapeutic avenues. Another new tactic involves using molecules that link splicing proteins to E3‐ubiquitin ligases, targeting them for proteasomal degradation. These can be either pre‐existing drugs or specifically designed proteolysis‐targeting chimeras, which are currently being used extensively as drug‐discovery tools to determine the function of proteins or the result of destroying the protein, but are quickly being developed as pharmaceutical agents. As discussed above, the discovery that the sulfonamide compound indisulam promotes the recruitment of the spliceosome‐associated protein RBM39 to the CUL4‐DCAF15 E3‐ubiquitin ligase^136^ and its degradation is associated with aberrant splicing and anticancer activity is quite exciting. Further developments in understanding the oncogenic mechanisms of splicing factor mutations may lead to novel approaches as well. For example, increased R‐loop formation is observed in spliceosome mutant leukemias and this has been shown to sensitize them to ATR inhibition.^147^ Moreover, unbiased genetic and chemical screening approaches in the context of spliceosome mutant cancers will be crucial for uncovering novel biological features, as well as uncovering therapeutically actionable targets.

The rapid development of ASO therapies in the treatment of neuromuscular diseases has paved way for this class of therapeutic approach. As delivery systems improve and safety in humans is further established, these therapies will expand in cancer clinical trials and using them to cause splice switching seems likely to be among the vanguard. Although still relatively nascent, newer nucleotide altering technologies such as gene editing by CRISPR‐Cas represent a very powerful and exciting strategy. The Cas13a enzyme, for example, allows for targeting and editing of RNA^148^, ^149^, ^150^, ^151^ and could represent an additional method to target critical splice sites to reverse aberrant splicing. As acquisition of spliceosome gene mutations appear to be the initiating events in clonal hematopoiesis and MDS pathogenesis, reversing the pathogenic splicing events by gene editing would ameliorate disease progression. This can also be envisioned to have clinical utility if the feasibility and safety can be established in humans.

Future clinical trials may also use a combination of the above agents (such as an ATR inhibitor with an SF3B complex inhibitor) or using the above agents in combination with nonsplicing specific therapies such as hypomethylating agents, apoptosis inducers (eg, BH‐3 mimetics), checkpoint blockade immunotherapies, or a number of mutation specific targeted agents in the case of patients with splicing factor mutation plus other mutations (eg, mutant IDH2 inhibitors). The future looks promising for finding means to address the very large need of novel therapies for the great number of patients with splicing factor mutant hematologic malignancies and for nonsplicing factor mutant malignancies that are dependent on splicing.

## CONFLICT OF INTEREST

The authors declare no conflict of interest and no competing interest.
